# Trefoil Factor 3 as a Biomarker for Peripheral Artery Disease

**DOI:** 10.3390/biom16060892

**Published:** 2026-06-17

**Authors:** Ben Li, Hamzah Khan, Farah Shaikh, Abdelrahman Zamzam, Ravel Raphael, Muzammil H. Syed, Rawand Abdin, Mohammad Qadura

**Affiliations:** 1 Division of Vascular Surgery, St. Michael’s Hospital, Unity Health Toronto, University of Toronto, 30 Bond Street, Toronto, ON M5B 1W8, Canada; benx.li@mail.utoronto.ca (B.L.);; 2 Department of Surgery, University of Toronto, Toronto, ON M5S 1A1, Canada; 3 Institute of Medical Science, University of Toronto, Toronto, ON M5S 1A1, Canada; 4 Temerty Centre for Artificial Intelligence Research and Education in Medicine (T-CAIREM), University of Toronto, Toronto, ON M5S 1A1, Canada; 5 Department of Medicine, McMaster University, Hamilton, ON L8S 4L8, Canada; 6 Heart, Vascular & Thoracic Institute, Cleveland Clinic Abu Dhabi, Abu Dhabi 112412, United Arab Emirates; 7 Li Ka Shing Knowledge Institute, St. Michael’s Hospital, Unity Health Toronto, University of Toronto, Toronto, ON M5B 1W8, Canada

**Keywords:** trefoil factor 3, biomarkers, diagnosis, prognosis, peripheral artery disease

## Abstract

Background: While trefoil factor 3 (TFF3) has been linked to cardiovascular disease, its role in peripheral artery disease (PAD) remains largely unexplored. In this prospective study, we assessed three pre-selected circulating biomarkers and found that TFF3 demonstrated the strongest association with the presence of PAD. Building on this finding, we integrated plasma TFF3 concentrations with clinical characteristics to construct predictive models aimed at identifying individuals with PAD and estimating their risk of major adverse limb events (MALE) over a two-year follow-up period. Methods: A total of 476 individuals were prospectively recruited, including 312 patients with PAD and 164 controls without PAD. At study entry, circulating concentrations of TFF3, oncostatin M (OSM), and brain-derived neurotrophic factor (BDNF) were quantified, and all participants were subsequently monitored for a two-year period. The primary endpoint was the occurrence of MALE within two years, comprising acute limb ischemia, major amputation, or lower extremity revascularization by either open surgical or endovascular approaches. PAD diagnosis served as the secondary outcome and was established by an ankle–brachial index (ABI) ≤ 0.9 or toe–brachial index (TBI) ≤ 0.67 in the presence of reduced or absent pedal pulses. For predictive model development, the cohort was randomly divided into training (70%) and testing (30%) sets. A random forest algorithm incorporating clinical variables and plasma TFF3 levels was developed and optimized using 10-fold cross-validation. Model discrimination was quantified using the area under the receiver operating characteristic curve (AUROC). For prognostic evaluation, patients were classified into low- and high-risk groups based on the optimal ROC-derived probability threshold of 0.60, and MALE-free survival between groups was assessed using Cox proportional hazards regression. Results: Among the three candidate biomarkers evaluated, only TFF3 demonstrated a significant association with PAD. Patients with PAD exhibited higher circulating TFF3 concentrations than those without PAD (7.27 ± 3.36 vs. 5.89 ± 2.67 pg/mL; *p* < 0.001), whereas OSM and BDNF showed no significant differences between groups. Over the two-year follow-up period, MALE occurred in 28 patients (9%). Predictive models combining plasma TFF3 measurements with clinical variables achieved strong performance for both PAD detection and 2-year MALE risk estimation, yielding AUROCs of 0.79 and 0.85, respectively. Furthermore, patients classified as high risk by the model experienced a significantly increased hazard of MALE during follow-up (HR 1.12, 95% CI 1.10–1.19; *p* = 0.003). Variable importance analysis revealed that TFF3 was the most influential predictor of MALE, followed by age and smoking history. Conclusions: Combining plasma TFF3 levels with readily available clinical characteristics enabled the development of a predictive model with good discriminatory ability for both PAD diagnosis and estimation of 2-year MALE risk. Such an approach may enhance risk stratification by identifying patients at elevated risk earlier in their disease course, thereby informing decisions related to vascular testing, referral for specialist evaluation, and implementation of targeted treatment strategies.

## 1. Introduction

Peripheral artery disease (PAD) is a common manifestation of systemic atherosclerotic disease affecting the lower extremity arterial circulation and is estimated to impact more than 200 million individuals globally [1,2]. Although PAD is associated with substantial risks of limb-related complications and death, it remains underrecognized and undertreated in routine clinical practice [3]. One challenge contributing to this burden is the lack of robust, clinically accessible biomarkers that can aid in disease detection, improve risk stratification, and guide therapeutic decision-making.

Trefoil factor 3 (TFF3), oncostatin M (OSM), and brain-derived neurotrophic factor (BDNF) are circulating proteins that have been demonstrated to be associated with cardiovascular diseases including cerebrovascular disease (CVD), coronary artery disease (CAD), and PAD [4,5,6]. The three candidate proteins evaluated in this study were selected based on prior evidence linking them to a range of cardiovascular disorders, suggesting that they may also have relevance in PAD [4,5,6]. Although these biomarkers have been shown to be associated with cardiovascular disease in previous studies, their utility for PAD diagnosis and prognostication remains largely unexplored [4,5,6].

Since PAD arises from a complex interplay of biological processes and pathophysiological pathways, we hypothesized that combining biomarker measurements with clinical variables would provide greater diagnostic and prognostic value than evaluating individual proteins alone [7]. The incorporation of circulating biomarker measurements with conventional demographic and clinical risk factors has the potential to improve risk assessment and enhance the prediction of adverse limb events in patients with PAD [8,9,10]. Therefore, the aim of this study was to evaluate candidate biomarkers associated with PAD and to integrate informative markers with clinical variables in predictive models for disease identification and prognostic stratification.

## 2. Materials and Methods

### 2.1. Ethics Approval

This study received approval from the Research Ethics Board at Unity Health Toronto, University of Toronto, Canada. Written informed consent was obtained from all participants before enrollment, and the study was conducted in accordance with the ethical principles set forth in the Declaration of Helsinki [11].

### 2.2. Design

This investigation was designed as a diagnostic and prognostic prediction study and was reported following the recommendations outlined in the TRIPOD+AI statement [12].

### 2.3. Cohort Recruitment

Participants were prospectively recruited from outpatient clinics at our institution between May 2018 and March 2021. The study population comprised both patients with PAD and individuals without evidence of the disease. PAD was defined by the presence of an ABI ≤ 0.9 or TBI ≤ 0.67 together with diminished or absent pedal pulses on clinical examination [13]. Participants were considered free of PAD if they had normal pedal pulses and vascular indices above these thresholds (ABI > 0.9 and TBI > 0.67) [13]. Individuals were excluded if they had experienced a recent acute cardiovascular or limb ischemic event, including acute coronary syndrome or acute limb ischemia, within the preceding three months. Patients with elevated cardiac troponin levels during this same period were also excluded from participation.

### 2.4. Baseline Variables

Baseline data collected for each participant included demographic characteristics, cardiovascular comorbidities, smoking history, and medication use. Recorded variables comprised age, sex, hypertension, dyslipidemia, diabetes mellitus, CAD, congestive heart failure (CHF), prior stroke, and current or previous tobacco use. Hypertension was defined as a diastolic blood pressure ≥80 mmHg, a systolic blood pressure ≥130 mmHg, or treatment with antihypertensive medication [14,15]. Dyslipidemia was defined by a triglyceride level >1.7 mmol/L, total cholesterol concentration >5.2 mmol/L, or use of lipid-lowering therapy [14,15]. Diabetes was identified by a hemoglobin A1c ≥6.5% or treatment with antidiabetic medication [14,15]. Medication data included the use of beta-blockers, statins, acetylsalicylic acid (ASA), oral antihyperglycemic agents, insulin, angiotensin-converting enzyme inhibitors (ACE-I) or angiotensin II receptor blockers (ARB), calcium channel blockers, and diuretics (hydrochlorothiazide or furosemide). Definitions of cardiovascular risk factors and medication categories were based on contemporary American College of Cardiology guideline recommendations [14,15].

### 2.5. Quantification of Plasma Protein Levels

Peripheral venous blood samples were obtained from the median cubital vein by trained phlebotomy personnel and collected in citrate-containing tubes. Following centrifugation, plasma was separated, aliquoted, and stored at −80 °C until analysis. Samples were not subjected to any freeze–thaw cycles prior to testing and were thawed at room temperature immediately before biomarker quantification. Plasma concentrations of TFF3, OSM, and BDNF were measured in duplicate using a commercially available Luminex assay platform (Bio-Techne, Minneapolis, MN, USA) [16]. These biomarkers were selected based on their proposed roles in atherosclerosis and previously reported associations with cardiovascular disease. Before sample acquisition, the MagPix system (Luminex Corp., Austin, TX, USA) [17] was calibrated according to manufacturer recommendations using Fluidics Verification and Calibration bead kits (Luminex Corp.) [18]. To minimize analytical variability, all specimens were processed during a single assay run. Assay precision was confirmed by maintaining both intra-assay and inter-assay coefficients of variation below 10%. A minimum of 50 beads per analyte were acquired for each sample, and data were analyzed using Luminex xPonent software version 4.3 [19].

### 2.6. Outcomes and Follow-Up

Participants underwent follow-up assessments in the outpatient clinic at 1 and 2 years after study enrollment. The primary endpoint was the occurrence of MALE within two years, defined as major lower extremity amputation above the ankle, lower extremity revascularization by either open surgical or endovascular techniques, or acute limb ischemia. Acute limb ischemia was defined as a sudden decrease in limb perfusion for less than 14 days resulting from arterial thrombosis or embolization. The secondary outcome was the presence of PAD, which was determined by vascular surgeons using clinical assessment and vascular investigations. PAD was defined as an ABI ≤ 0.9 or TBI ≤ 0.67 in conjunction with diminished or absent pedal pulses [13]. Because all MALE outcomes occurred exclusively among patients with PAD, prognostic analyses were restricted to the PAD subgroup. In contrast, diagnostic modeling was performed using the entire study population, including both participants with and without PAD.

### 2.7. Model Training and Assessment

Random forest was selected as the predictive modeling approach for this study. As an ensemble machine learning technique, random forest generates predictions by aggregating the outputs of numerous decision trees [20]. Individual decision trees recursively partition data into increasingly homogeneous subgroups based on predictor variables, enabling classification or prediction of a specified outcome [21]. Because random forest is a non-parametric method, it can effectively learn complex relationships in high-dimensional data [21]. This method was selected because of its extensive use in prior research and its consistently strong performance in a broad range of clinical prediction and healthcare analytics applications [22,23,24].

The study cohort was randomly partitioned into training (70%) and testing (30%) datasets. Random forest algorithms were developed using 10-fold cross-validation to model both PAD diagnosis and the risk of 2-year MALE. Predictor variables included plasma TFF3 concentrations in addition to demographic characteristics, cardiovascular risk factors, comorbid conditions, and medication use. Specifically, model inputs comprised sex, age, smoking history (current or former), dyslipidemia, diabetes, hypertension, CAD, prior stroke, CHF, and treatment with oral antihyperglycemic agents, insulin, beta-blockers, statins, ACE-I/ARB therapy, calcium channel blockers, ASA, and hydrochlorothiazide or furosemide. Following model development, performance was assessed in the independent testing dataset using the area under the receiver operating characteristic curve (AUROC) as the primary measure of discrimination [25]. Diagnostic models were trained and evaluated using the full cohort of participants with and without PAD, whereas prognostic analyses were limited to patients with PAD because all MALE outcomes occurred within this subgroup. To identify the variables contributing most strongly to model predictions, feature importance was quantified using the gain metric, which estimates the relative contribution of each predictor to overall model performance [26].

### 2.8. Statistical Analysis

Continuous variables are presented as means ± SD, whereas categorical variables are reported as frequencies and percentages. Baseline differences between study groups were evaluated using independent *t*-tests for continuous variables and chi-square tests for categorical variables. Comparisons of circulating biomarker concentrations between participants with and without PAD were performed using independent *t*-test (if normally distributed) or Mann–Whitney U test (if non-normally distributed). Biomarkers demonstrating significant associations with PAD were subsequently selected for inclusion in predictive model development. Rates of 2-year clinical events were compared using the chi-square test. Model performance was evaluated for both PAD diagnosis (PAD vs. non-PAD) and prediction of 2-year MALE among patients with PAD. Discriminatory ability was quantified using the AUROC as the primary performance metric. For prognostic analyses, an optimal probability threshold of 0.60 was identified within the training dataset using the Youden Index, a widely accepted measure that maximizes the combined sensitivity and specificity of a predictive model [27]. This threshold was then applied without modification to the testing dataset. To evaluate the clinical relevance of risk predictions, patients were categorized as low- or high-risk for MALE and compared using Kaplan–Meier survival analysis. The relationship between model-derived risk classification and MALE was further examined using multivariable Cox proportional hazards regression adjusted for all baseline variables. These analyses were performed to determine whether the model could effectively discriminate between patients with differing risks of adverse limb outcomes during follow-up. Missing data accounted for less than 5% of observations; therefore, complete case analysis was performed. Sensitivity analyses demonstrated no meaningful evidence of selection bias arising from missing data. To account for multiple comparisons, a Bonferroni correction was applied to set statistical significance. All statistical analyses were conducted using SPSS version 23 (SPSS Inc., Chicago, IL, USA) [28].

## 3. Results

### 3.1. Cohort

The study cohort comprised 476 participants, including 312 patients with PAD and 164 individuals without PAD. Compared with those without PAD, patients with PAD were significantly older (71 ± 10 vs. 65 ± 12 years, *p* < 0.001) and had a greater prevalence of cardiovascular risk factors and comorbid conditions, including dyslipidemia (84% vs. 61%, *p* < 0.001), hypertension (82% vs. 59%, *p* < 0.001), diabetes (42% vs. 21%, *p* < 0.001), prior stroke (16% vs. 8%, *p* = 0.011), CAD (38% vs. 21%, *p* < 0.001), and current or former smoking (80% vs. 64%, *p* = 0.002). Consistent with their higher cardiovascular risk profile, patients with PAD were also more frequently treated with preventive cardiovascular therapies, including statins (73% vs. 57%, *p* < 0.001), ASA (80% vs. 60%, *p* < 0.001), ACE-I/ARB therapy (66% vs. 45%, *p* = 0.001), and beta-blockers (41% vs. 30%, *p* = 0.001) (Table 1).

### 3.2. Plasma Proteins

Among the three biomarkers evaluated, only TFF3 demonstrated higher circulating concentrations in patients with PAD than in those without PAD: 7.27 [SD 3.36] vs. 5.89 [SD 2.67] pg/ml, *p* < 0.001 (Table 2). TFF3 was therefore included in further analyses.

### 3.3. Adverse Events

During the 2-year follow-up period, all MALE outcomes were observed exclusively in patients with PAD, with no adverse limb events occurring among participants without PAD. This included 28 patients who developed MALE (9%), 19 patients who required a lower extremity vascular intervention (6%), and 17 patients who underwent a major amputation (5%). No episodes of acute limb ischemia were observed during follow-up (Table 3). Elevated TFF3 levels were significantly associated with an increased risk of adverse limb outcomes, including MALE (HR 1.12, 95% CI 1.10–1.19; *p* = 0.003), lower extremity vascular intervention (HR 1.16, 95% CI 1.06–1.27; *p* = 0.001), and major amputation (HR 1.11, 95% CI 1.01–1.24; *p* = 0.017) (Table 4).

### 3.4. Model Performance

**Figure 1:**
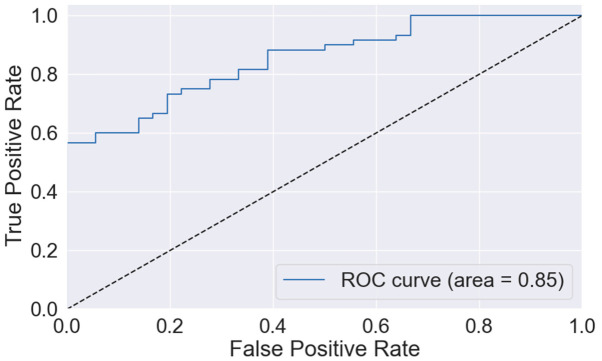
Receiver operating characteristic (ROC) curve demonstrating the performance of the random forest model incorporating clinical variables and trefoil factor 3 (TFF3) for predicting 2-year major adverse limb events (MALE) among patients with PAD in the testing dataset.

**Figure 2:**
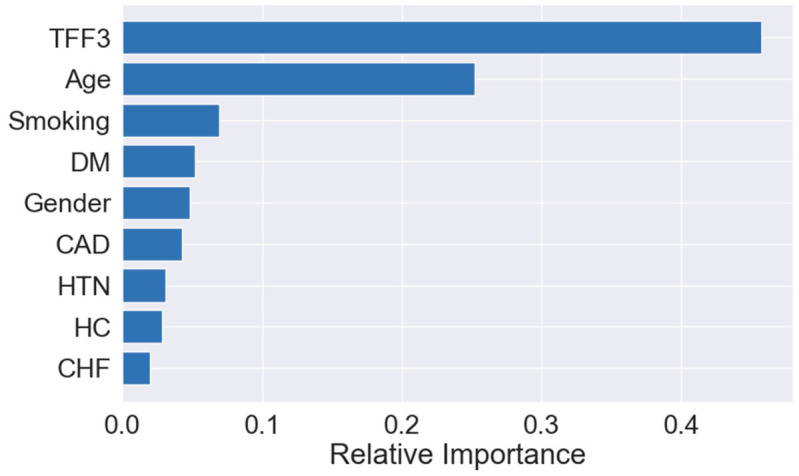
Variable importance analysis showing the relative contribution (gain) of trefoil factor 3 (TFF3) and clinical predictors included in the random forest model for estimating the risk of 2-year major adverse limb events (MALE) among patients with peripheral artery disease. Abbreviations: trefoil factor 3 (TFF3), diabetes mellitus (DM), coronary artery disease (CAD), hypercholesterolemia (HC), congestive heart failure (CHF), and hypertension (HTN).

**Figure 3:**
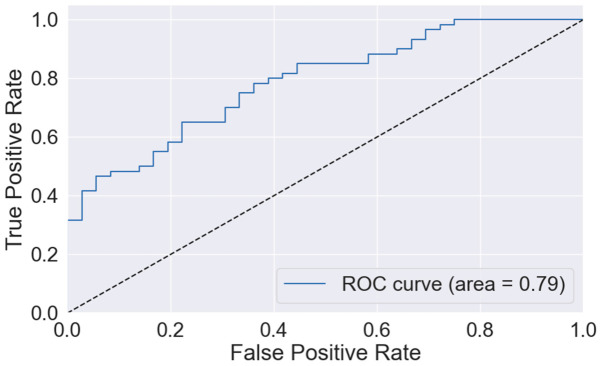
Receiver operating characteristic (ROC) curve demonstrating the diagnostic performance of the random forest model integrating clinical characteristics and trefoil factor 3 (TFF3) for identifying peripheral artery disease in the testing cohort.

A random forest model incorporating plasma TFF3 concentrations and clinical variables demonstrated strong discrimination for predicting 2-year MALE, achieving an AUROC of 0.85 (Figure 1). Feature importance analysis identified TFF3 as the most influential predictor, followed by age and current/past smoking status (Figure 2). For PAD diagnosis, the model also performed well, yielding an AUROC of 0.79 (Figure 3).

### 3.5. Model-Driven Risk Stratification

**Figure 4:**
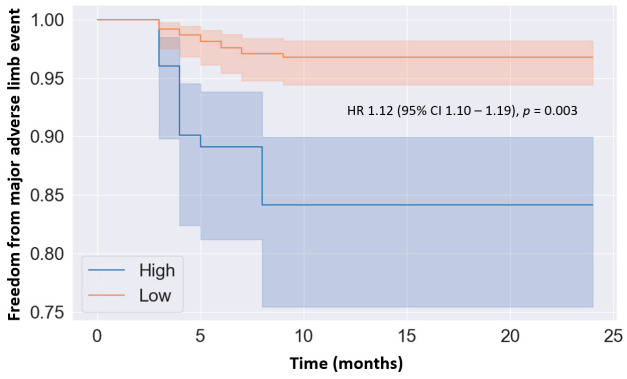
Kaplan–Meier curves depicting freedom from major adverse limb events (MALE) among patients classified as low-risk or high-risk by the random forest prognostic model. Abbreviations: HR (hazard ratio), CI (confidence interval).

Using Youden’s Index, the optimal cut-point for predicting 2-year MALE was identified as a model probability score of 0.60. Based on this threshold, patients were stratified into high- and low-risk groups. Kaplan–Meier survival analysis demonstrated a significantly greater incidence of MALE among individuals classified as high risk, with reduced MALE-free survival observed throughout the 2-year follow-up period compared with the low-risk group (HR 1.12, 95% CI 1.10–1.19; *p* = 0.003) (Figure 4).

## 4. Discussion

### 4.1. Summary of Findings

In this study, we identified TFF3 as a circulating biomarker associated with PAD and incorporated plasma TFF3 concentrations into predictive models alongside clinical characteristics to assess both disease presence and future limb-related risk. A key finding was that, among the three candidate biomarkers evaluated, TFF3 was the only marker that differed significantly between patients with and without PAD, with higher circulating levels observed in the PAD cohort. In addition, integration of TFF3 with clinical variables enabled the development of prediction models that demonstrated good discriminatory performance for both PAD diagnosis and 2-year MALE prediction. Feature importance analysis demonstrated that the most important predictor for PAD prognosis was TFF3 levels, highlighting the importance of biomarkers in predicting PAD outcomes. The strong diagnostic and prognostic associations observed for TFF3 support the need for further laboratory, translational, and clinical investigations to clarify its mechanistic links to PAD. Such work may provide insight into disease biology while informing future biomarker-driven approaches to diagnosis and treatment. Our prognostic algorithm successfully categorized patients according to their risk of subsequent limb-related complications. Survival analysis demonstrated a significantly higher incidence of MALE among individuals assigned to the high-risk category compared with those deemed low-risk over the 2-year follow-up period (HR 1.12). These results suggest that the model may serve as a valuable tool for clinical risk assessment, enabling the identification of patients who could benefit from enhanced monitoring and more aggressive therapeutic strategies.

### 4.2. Comparison to Existing Literature

Ross and colleagues (2019) developed machine learning models using electronic health record data to predict the occurrence of Major Adverse Cardiac and Cerebrovascular Events (MACCE) among patients with PAD [29]. Their models were derived from retrospectively collected data, including International Classification of Diseases (ICD)-9 codes, Common Procedural Terminology (CPT) codes, medication records, and a range of clinical characteristics, with the goal of forecasting MACCE occurring more than 30 days after PAD diagnosis [29]. The final model demonstrated good discriminative ability, achieving an AUROC of 0.81 [29]. Nevertheless, the study did not incorporate circulating biomarkers, despite growing evidence that biomarker information can provide important diagnostic and prognostic insights in PAD [4,5,6]. To address this gap, we integrated biomarker measurements into our machine learning framework. By combining plasma TFF3 concentrations with clinical variables, our model achieved an AUROC of 0.85 for predicting 2-year MALE. These findings indicate that the addition of biomarker data may improve predictive performance beyond that attainable with clinical variables alone. Furthermore, while Pesau et al. (2025) reported an association between serum TFF3 levels and all-cause mortality in PAD patients [4], our work expands upon these observations by demonstrating the value of incorporating TFF3 into machine learning models designed to predict limb-specific outcomes. This approach highlights the potential clinical utility of TFF3 as a biomarker for risk stratification and prognostication in PAD [4].

### 4.3. Explanation of Findings

Several biological mechanisms may help explain these observations. Notably, patients with PAD in our cohort exhibited significantly higher circulating TFF3 levels than individuals without PAD. Furthermore, TFF3 was among the most influential variables in our predictive models, contributing substantially to the identification of PAD and the estimation of subsequent adverse limb events. TFF3 is a small-molecule peptide (6 kD) formed by the trefoil domain consisting of disulfide bonds [30]. It was identified more than 3 decades ago and is typically secreted by goblet cells and expressed by mucous epithelial [30]. It has been found to be expressed in various organ systems including the heart, muscle, and liver [30]. TFF3 has also been demonstrated to be associated with angiogenesis and is involved in wound healing and cellular migration [31]. Others have linked TFF3 to cardiovascular diseases secondary to its pro-atherogenic and inflammatory effects [4]. Specifically, TFF3 has been shown to be involved in the upregulation of cyclooxygenase-2 resulting in an increase in prostaglandin synthesis and promotion of systemic inflammation [32]. Recently, TFF3 has been demonstrated to be associated with adverse cardiovascular events in patients with heart failure [33]. Collectively, these observations provide biological plausibility for the involvement of TFF3 in the development and progression of PAD. The non-PAD group included patients with other vascular diseases who attended outpatient vascular clinics at our institution. This includes patients with carotid, aortic, or venous disease. While these patients do not have PAD, their other vascular diseases may have contributed to higher concentrations of OSM, TFF3, and BDNF compared to the general population. Second, we observed a substantial burden of adverse limb outcomes, with approximately 10% of patients experiencing MALE during the 2-year follow-up period. This observation underscores the importance of developing more effective risk assessment strategies to identify vulnerable patients earlier, enabling prompt management and potentially reducing the incidence of limb-related adverse events. Third, the strong performance of our predictive model may be attributable, in part, to the advantages of advanced machine learning methods. Unlike conventional statistical approaches such as logistic regression, which assume a linear relationship between predictor variables and the log odds of an outcome, machine learning algorithms can capture complex, nonlinear interactions and patterns within the data, potentially enhancing predictive accuracy [34,35]. The ability to model complex relationships is particularly advantageous in healthcare datasets, where clinical outcomes are often determined by numerous interrelated patient characteristics and biological processes [36]. Machine learning methods offer several benefits over conventional statistical approaches, including the capacity to automate pattern recognition, capture nonlinear associations, and generate highly accurate predictions [34,35]. These strengths may be especially important in biomarker-based prediction models, as circulating proteins participate in diverse biological pathways and may exert synergistic or interactive effects on disease development and progression [37]. In the present study, the strong performance of the random forest algorithm may be attributable to its ensemble-based architecture, which combines the predictions of numerous decision trees to improve predictive accuracy [38]. This approach can reduce model variance, improve generalizability, and limit overfitting while maintaining the ability to analyze complex datasets [38]. Our results further support the value of integrating biomarker information into predictive models, as the inclusion of TFF3 alongside clinical characteristics yielded strong diagnostic and prognostic performance. In addition to circulating biomarkers, previous studies by Wen et al. (2024) and Liang et al. (2019) have demonstrated that lifestyle factors and medical therapies may influence the development and progression of vascular disease, highlighting the multifactorial nature of cardiovascular risk [39,40]. The complex pathophysiology of PAD involves multiple interacting biological mechanisms, making accurate diagnosis and prognostication inherently challenging. Accordingly, previous studies have emphasized the value of integrative approaches that incorporate diverse sources of clinical and biological information to improve risk assessment in PAD [41]. Consistent with this concept, our findings demonstrate that combining circulating biomarker data with clinical characteristics within advanced predictive modeling frameworks can yield highly effective tools for PAD diagnosis and prognostication.

### 4.4. Clinical Implications

Our predictive models have several potential applications in clinical practice. One important use may be the identification of individuals with previously unrecognized PAD, particularly in primary care settings. By integrating the algorithm into routine clinical assessments, family physicians could estimate a patient’s likelihood of having PAD and identify those who may benefit from further vascular investigation [42]. Patients deemed at increased risk could subsequently undergo confirmatory testing, such as arterial duplex ultrasound, to evaluate lower extremity perfusion and establish a definitive diagnosis [43]. Beyond diagnosis, the model may also support prognostic assessment among patients with established PAD. Individuals classified as lower risk could continue to receive longitudinal management in the primary care setting, with emphasis on guideline-directed medical therapy and risk factor modification, including antiplatelet therapy, statin use, smoking cessation, and exercise interventions [44]. Conversely, patients identified as having an elevated risk of MALE may warrant referral to a vascular specialist for further evaluation and consideration of more intensive treatment strategies [45]. Within specialty care, the algorithm may serve as an adjunct to clinical judgment by helping identify patients most likely to benefit from additional vascular imaging to characterize disease extent and anatomy [46], intensified medical therapy such as low-dose rivaroxaban [47], or revascularization procedures aimed at limb preservation in selected high-risk individuals [48,49,50].

### 4.5. Limitations

Several limitations should be considered when interpreting our findings. First, this investigation was conducted at a single institution, which may limit the generalizability of the results. Validation in external cohorts from diverse healthcare settings may help determine the broader applicability of the predictive models. Second, outcome assessment was restricted to a 2-year follow-up period. Given the chronic and progressive nature of PAD, studies with longer follow-up durations are needed to better characterize the long-term prognostic performance of the algorithm. Thirdly, the risk stratification analyses were performed within the same cohort used for model development and evaluation. Consequently, these findings should be viewed as an internal assessment of prognostic utility. External validation in independent cohorts is needed to further establish the generalizability and clinical applicability of the model. Fourthly, although the Cox proportional hazards analysis was adjusted for clinically relevant covariates, the number of observed MALE outcomes was relatively small. Consequently, the adjusted hazard ratio estimates may lack precision and should be considered exploratory and interpreted cautiously. Future studies with larger sample sizes and greater numbers of outcome events are needed to confirm the observed associations. Additionally, the probability threshold used for risk stratification was derived within the training dataset and has not been externally validated. Future studies are needed to confirm its generalizability across different patient populations. Finally, the biomarkers evaluated in this study are currently used primarily within research environments. Additional translational, clinical, and implementation studies are required to determine their feasibility, cost-effectiveness, and practical value for routine PAD screening, risk stratification, and prognostication.

## 5. Conclusions

In this study, we identified TFF3 as a biomarker associated with PAD and demonstrated that integrating plasma TFF3 levels with clinical variables can produce accurate predictive models for both PAD diagnosis and prognostication. These models showed strong discriminatory performance and may support earlier identification of patients at elevated risk for adverse limb outcomes. By facilitating risk stratification, the algorithm could help direct higher-risk individuals toward more comprehensive vascular evaluation, enhanced surveillance, and intensified medical management aimed at mitigating future complications. In addition, our findings underscore the need for further mechanistic and translational investigations exploring the role of TFF3 in the initiation and progression of PAD. A deeper understanding of the biological pathways linking TFF3 to vascular disease may not only advance knowledge of PAD pathophysiology but also inform the development and refinement of biomarker-based approaches for diagnosis, prognostication, and targeted therapy.

## Figures and Tables

**Table 1:** Baseline characteristics.

	Non-PAD (*n* = 164)	PAD (*n* = 312)	*p*
Age, mean (SD)	65 (12)	71 (10)	<0.001
Female sex	67 (41)	109 (35)	0.204
Hypertension	96 (59)	257 (82)	<0.001
Dyslipidemia	100 (61)	263 (84)	<0.001
Diabetes	34 (21)	131 (42)	<0.001
Past smoking	71 (43)	171 (55)	0.001
Current smoking	35 (21)	78 (25)	0.002
Congestive heart failure	4 (2)	11 (4)	0.519
Coronary artery disease	34 (21)	118 (38)	<0.001
Previous stroke	13 (8)	51 (16)	0.011
Acetylsalicylic acid	99 (60)	251 (80)	<0.001
Statin	93 (57)	229 (73)	<0.001
ACE-I/ARB	74 (45)	216 (66)	0.001
Beta blocker	50 (30)	134 (41)	0.001
Calcium channel blocker	34 (21)	82 (25)	0.079
Hydrochlorothiazide or furosemide	17 (10)	41 (13)	0.190
Oral antihyperglycemic agent	8 (5)	24 (8)	0.201
Insulin	6 (4)	22 (7)	0.255

Values reported as *n* (%) unless otherwise indicated. Abbreviations: PAD (peripheral artery disease), SD (standard deviation), ACE-I (angiotensin-converting enzyme inhibitor), and ARB (angiotensin II receptor blocker).

**Table 2:** Plasma concentrations of proteins in patients with and without peripheral artery disease.

	Non-PAD (*n* = 164)		PAD (*n* = 312)		
	Mean	Standard Deviation	Mean	Standard Deviation	*p*
TFF3	5.89	2.67	7.27	3.36	<0.001
OSM	574.41	217.54	625.04	266.23	0.051
BDNF	13.93	13.38	15.44	13.15	0.06

Protein concentrations reported in pg/ml. Abbreviations: trefoil factor 3 (TFF3), oncostatin M (OSM), brain-derived neurotrophic factor (BDNF).

**Table 3:** Adverse limb events over 2 years of follow-up.

	PAD (*n* = 312)	Non-PAD (*n* = 164)	*p*
Major adverse limb event	28 (9)	0 (0)	0.001
Major amputation	17 (5)	0 (0)	0.002
Vascular intervention	19 (6)	0 (0)	0.001
Acute limb ischemia	0 (0)	0 (0)	N/A

Values reported as *n* (%) unless otherwise indicated. Abbreviation: PAD (peripheral artery disease).

**Table 4:** Adjusted associations between trefoil factor 3 and two-year major adverse limb events.

	Hazard Ratio [95% CI]	*p*-Value
Major adverse limb event	1.12 [1.10–1.19]	0.003
Major amputation	1.11 [1.01–1.24]	0.017
Vascular intervention	1.16 [1.06–1.27]	0.001

## Data Availability

The original contributions presented in the study are included in the article; further inquiries can be directed to the corresponding author.
